# Review of the leafhopper subgenus Pediopsoides (Sispocnis) (Hemiptera, Cicadellidae, Eurymelinae, Macropsini) with description of two new species from China

**DOI:** 10.3897/zookeys.855.33591

**Published:** 2019-06-13

**Authors:** Hu Li, Juan Li, Ren-Huai Dai

**Affiliations:** 1 Shaanxi Key Laboratory of Bio-resources, School of Biological Science & Engineering, Shaanxi University of Technology, Hanzhong, Shaanxi, 723000 P.R. China Shaanxi University of Technology Hanzhong China; 2 Institute of Entomology of Guizhou University, The Provincial Key Laboratory for Agricultural Pest Management of Mountainous Region, Guiyang, Guizhou, 550025 P.R. China Institute of Entomology of Guizhou University Guiyang China

**Keywords:** Auchenorrhyncha, morphology, new type, taxonomy

## Abstract

The leafhopper subgenus Pediopsoides (Sispocnis) Anufriev, 1967 is reviewed and the type species is fixed as *Bythoscopuskogotensis* Matsumura, 1912. Six valid species of the subgenus are recognized including two new species described and illustrated here, Pediopsoides (Sispocnis) rectus Li, Li & Dai, **sp. nov.** and P. (S.) triangulus Li, Li & Dai, **sp. nov.** from Sichuan Province of Southwestern China. Additionally P. (S.) heterodigitatus Dai & Zhang, 2009 is proposed as a junior synonym of P. (S.) aomians (Kuoh, 1981) based on examination of many specimens. A key to species of the subgenus is also provided for identification.

## Introduction

The leafhopper genus *Pediopsoides* belonging to the subfamily Eurymelinae, tribe Macropsini (Dietrich and Thomas 2018) of Cicadellidae (Hemiptera: Auchenorrhyncha) was originally established by Matsumura (1912) with *Pediopsoidesformosanus*Matsumura, 1912 as its type species, and divided into four subgenera based on the features of the facial and tegminal proportions, and the male genitalia by Hamilton (1980). *Sispocnis* is just one of the four subgenera of *Pediopsoides* as proposed by Hamilton (1980), and was originally placed in the genus *Oncopsis*, erected by Anufriev (1967) for *Bythoscopusjuglans* Matsumura, 1912 (misidentified type species). Later, Viraktamath (1981) added a new species, Pediopsoides (Sispocnis) sharmai from India; Dai and Zhang (2009) described two new species, P. (S.) dilatus and P. (S.) heterodigitatus from China, proposed a new combination, P. (S.) aomians from the genus *Oncopsis*, and revealed two new synonyms: *Digitalis* Liu & Zhang, 2002 as a synonym of *Pediopsoides* and *Digitalisstriolatus* Liu & Zhang, 2002 as a junior synonym of P. (S.) aomians (Kuoh, 1981). So far, five species of Pediopsoides (Sispocnis) are known from the world including four species recorded in China.

In our marcopsine collection from Sichuan Province of China, two new species of Pediopsoides (Sispocnis) are recognized, and their illustrated descriptions are provided in the present paper. Based on the examination of the specimens, the subgenus Pediopsoides (Sispocnis) is simultaneously reviewed and a key is given for identification. It is revealed that P. (S.) heterodigitatus Dai & Zhang, 2009 is a junior synonym of P. (S.) aomians. To date, six species of the subgenus Pediopsoides (Sispocnis) are known, five of which occur in China.

## Materials and methods

Adult specimens collected by sweep net were used for examination, description, illustration and imaging. The habitus images of adults were obtained with an Olympus SZX7 microscope mounted with a Canon EOS 550D camera.

The morphological terminologies and the higher classification system follow Hamilton (1980). The body length is measured from the apex of the head to the end of the forewings and is given in millimeters (mm).

The type materials of the new species are deposited in the Museum of Zoology and Botany, Shaanxi University of Technology, Hanzhong, China (SUHC). Other examined specimens are deposited in the Institute of Entomology, Guizhou University, Guiyang, China (GUGC).

## Systematics

### Genus Pediopsoides

Taxon classificationAnimaliaHemipteraCicadellidae

Matsumura, 1912

Pediopsoides Matsumura, 1912: 305

#### Type species.

*Pediopsoidesformosanus* Matsumura, 1912

### Subgenus Pediopsoides (Sispocnis)

Taxon classificationAnimaliaHemipteraCicadellidae

Anufriev, 1967

Oncopsis (Sispocnis) Anufriev, 1967: 174
Pediopsoides (Sispocnis) Hamilton, 1980: 897

#### Type species.

*Bythoscopuskogotensis* Matsumura, 1912, new designation.

The type species is fixed here under Article 70.3 of the ICZN as *Bythoscopuskogotensis* Matsumura, 1912, misidentified as *Bythoscopusjuglans* Matsumura, 1912 in the original designation of *Sispocnis* by Anufriev (1967).

#### Distribution.

Palaearctic and Oriental Regions.

#### Diagnosis.

Pediopsoides (Sispocnis) is well known by the following features: face including eyes is clearly wider than long; the stripes on pronotum are usually transverse and weakly obscure, forewing has anteapical cells of variable number (2 or 3), male pygofer ventral processes generally bifid to multifid and twisted inward; and the dorsal connective usually carries a strongly developed process from its inner ventral margin.

#### Remarks.

*Sispocnis* was originally established as a subgenus under the genus *Oncopsis* by Anufriev (1967) for *Bythoscopusjuglans* Matsumura, 1912 which was designated as the type species. Hamilton (1980) revised the status of *Sispocnis* and proposed it as a subgenus within *Pediopsoides*. Hamilton (1980) also recognized Anufriev’s (1967) identification of *B.juglans* Matsumura as a misidentification, and provisionally considered Oncopsis (Sispocnis) kurentsovi Anufriev as the type of the subgenus. Based on examination of the type specimens of Matsumura (1912) and Anufriev (1977), Okudera (2014) considered O. (S.) kurentsovi Anufriev as a junior synonym of *B.kogotensis* Matsumura, and recognized misidentifications of this species as *B.juglans* by Anufriev (1967) and Anufriev and Emeljanov (1988). Additionally, Okudera (2014) considered *B.juglans* Matsumura as a junior synonym of *Oncopsisnitobei* (Matsumura, 1912).

Herein, it is necessary to clarify the type species for the subgenus. Following the provisions of Article 70.3.2 of the International Code of Zoological Nomenclature (ICZN, 1999), the type species is fixed here as *Bythoscopuskogotensis* Matsumura, 1912, misidentified as *Bythoscopusjuglans* Matsumura, 1912 in the original designation by Anufriev (1967). Thus, Pediopsoides (Sispocnis) remains as a valid subgenus.

### Pediopsoides (Sispocnis) aomians

Taxon classificationAnimaliaHemipteraCicadellidae

(Kuoh, 1981)

Figs 1–26

Oncopsis aomians Kuoh, 1981: 201
Digitalis striolatus Liu & Zhang, 2002: 175 (synonym by Dai and Zhang 2009)
Pediopsoides (Sispocnis) aomians , Dai & Zhang 2009: 28
Pediopsoides (Sispocnis) heterodigitatus Dai & Zhang, 2009: 31. syn. n.

#### Material examined.

GUGC: 1 ♂, CHINA: Shaanxi Province, Mei County, Taibaishan National Natural Reserve, 17.vii.2012, collected by Fan Zhi-Hua; 1 ♀, CHINA: Sichuan Province, Tibetan Autonomous Prefecture of Garzê, Kangding County, 31.vii.2012, collected by Fan Zhi-Hua; 2 ♀♀, CHINA: Yunnan Province, Diqing Tibetan Autonomous Prefecture, Shangri-la, 08.viii.2012, collected by Fan Zhi-Hua; 1 ♂, CHINA: Qinghai Province, Tu Autonomous County of Huzhu, Beishan Forest Farm, 2685 m, 17.viii.2008, collected by Song Qiong-Zhang; 2 ♂♂, CHINA: Qinghai Province, Datong Hui & Tu Autonomous County, Black Spring Reservoir, 3000 m, 09.vii.2007, collected by Chen Xiang-Sheng; 1 ♂ 7 ♀♀, CHINA: Sichuan Province, Tibetan Qiang Autonomous Prefecture of Ngawa, Songpan County, Huanglong Temple, 22.viii.1994, collected by Du Yu-Zhou; 5 ♀♀, CHINA: Yunnan Province, Nujiang of the Lisu Autonomous Prefecture, Lushui City, PianMa Town, 17.viii.2001, collected by Yang Mao-Fa; 2 ♂♂ 4 ♀♀, CHINA: Shanxi Province, Xinzhou City, Ningwu County, Luyashan National Natural Reserve, 18.viii.2011, collected by Li Hu, Fan Zhi-Hua & Yu Xiao-Fei. SUHC: 4 ♂♂ 4 ♀♀, CHINA: Sichuan Province, Tibetan Autonomous Prefecture of Garzê, Luding County, Moxi Town, Hailuogou, 3600 m above sea level, 12.viii.2015, collected by Zhan Hong-Ping; 1 ♂ 1 ♀, CHINA: Sichuan Province, Tibetan Autonomous Prefecture of Garzê, Daocheng County, Sangdui Town, 4100 m above sea level, 15.viii.2015, collected by Zhan Hong-Ping; 1 ♂ 1 ♀, CHINA: Sichuan Province, Tibetan Autonomous Prefecture of Garzê, Luding County, Minya Konka, Yajiageng, 3800 m above sea level, 13.viii.2015, collected by Zhan Hong-Ping; 1 ♂, CHINA: Sichuan Province, Tibetan Autonomous Prefecture of Garzê, Luding County, Xindianzi, 2845 m above sea level, 13.viii.2015, collected by Zhan Hong-Ping.

#### Description.

**Figures 1–26.**
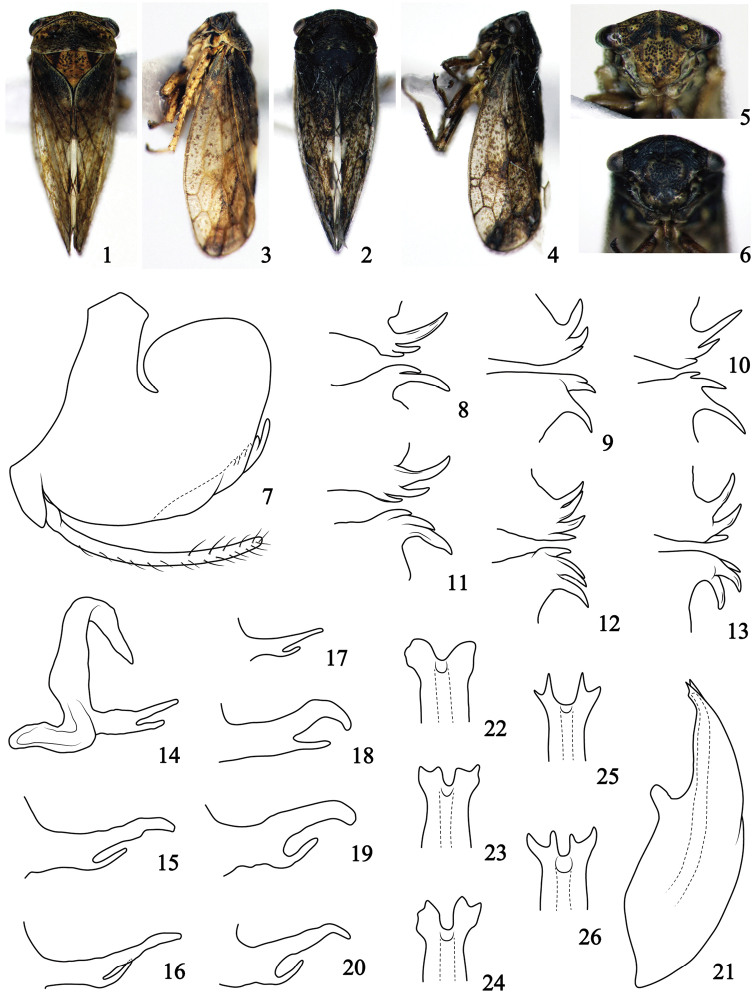
Pediopsoides (Sispocnis) aomians (Kuoh) **1, 2** male habitus, dorsal view **3, 4** male habitus, lateral view **5, 6** face **7** male pygofer and subgenital plate, lateral view **8–13** pygofer ventral processes (from several specimens), ventrocaudal view **14** dorsal connective, lateral view **15–20** process of dorsal connective from inner ventral margin, lateral view **21** aedeagus, lateral view **22–26** apex of aedeagus, ventral view.

*Body color* (Figs 1–6). Yellow brown to dark brown or black, usually densely marked with darker maculae. Pronotum (Figs 1, 2) generally with 4–6 shallow yellowish or brown spots on posterior margin. Scutellum (Figs 1, 2) with black triangular spots on lateral corners, or evenly black.

*Body appearance* (Figs 1–6). Stout and wedge-shaped. Head (Figs 1, 2) with frontal margin sinuated slightly. Face (Figs 5, 6) with stripes and punctures; frons clearly with longitudinal carina in middle, and oblique striations from carina to lateral margins; lower parts of postclypeus tumid. Pronotum (Figs 1, 2) 2.5 times wider than long, scutellum 1.2 times longer than that of pronotum. Forewing (Figs 3, 4) with three anteapical and four apical cells, and occasionally with additional cross-vien(s).

*Male genitalia*. Pygofer (Figs 7–13) basally broad, ventrocaudal margin with multifid and inturned process usually with 2–4 figure-like or spine-like branches of different size. Dorsal connective (Figs 14–20) strongly developed, S-shaped, with bifurcate various process from inner ventral margin with dorsal branch usually larger, longer and twisted ventrad and ventral branch small, short and dorsally bent. Aedeagus (Figs 21–26) broad basally, shaft margins parallel or slightly sinuate at ventral aspect, apical dorsal margin of shaft elongate to two lobes (Fig. 22) and usually bifid at varying levels (Figs 23–26) with each lobe.

#### Distribution.

China (Shaanxi, Sichuan, Qinghai, Shanxi, Gansu and Yunnan).

#### Remarks.

After examination of many specimens, the morphological diversity of P. (S.) aomians in body color and male genital structures is now better understood. Pediopsoides (S.) aomians can be distinguished from other members of Pediopsoides (Sispocnis) largely by the multifid (2–4) figure-like or spine-like process on distal caudoventral margin of male pygofer, the bifurcate process from inner ventral margin of the dorsal connective, and the unique structure of the aedeagus. The original description of P. (S.) heterodigitatus was based on only one male from Yunnan Province of China and the shape of male genitalia according to the description of that species falls well within the variation of P. (S.) aomians as interpreted here. It is proposed herein as a junior synonym of P. (S.) aomians.

### Pediopsoides (Sispocnis) dilatus

Taxon classificationAnimaliaHemipteraCicadellidae

Dai & Zhang, 2009

Pediopsoides (Sispocnis) dilatus Dai & Zhang, 2009: 31

#### Material examined.

2 ♂♂, CHINA: Qinghai Province, Datong Hui & Tu Autonomous County, Black Spring Reservoir, 3000 m, 09.vii.2007, collected by Chen Xiang-Sheng; 1 ♀, CHINA: Sichuan Province, Tibetan Autonomous Prefecture of Garzê, Kangding County, Scenic Spot of Kangding Love Song (= Mugecuo), 3600–3800 m, 30.viii.2008, collected by Yang Mao-Fa; 14 ♀♀, CHINA: Guizhou Province, Zunyi City, Suiyang County, Kuankuoshui National Natural Reserve, Chachang/Shuiku, 03-09.vi.2010, collected by Li Hu, Dai Ren-Huai & Xing Ji-Chun; 2 ♀♀, CHINA: Guizhou Province, Qiandongnan Miao & Dong Autonomous Prefecture, Leishan County, Leigongshan National Natural Reserve, 05.vii.2011, collected by Zheng Wei-Bin; 1 ♀, CHINA: Shandong Province, Qingdao City, Laoshan Mountain, 17.viii.2011, collected by Chang Zhi-Min.

#### Distribution.

China (Sichuan, Qinghai, Guizhou, Shandong and Xizang).

#### Remarks.

This species can be easily recognized by the teeth on the caudoventral margin of the male pygofer, the aedeagal shaft broadened gradually from the base to the end, the lateral triangular expansions on shaft apex, and the dorsal connective with the process from the inner ventral margin weakly sclerotized, slender, short and unbranched.

### Pediopsoides (Sispocnis) kogotensis

Taxon classificationAnimaliaHemipteraCicadellidae

(Matsumura, 1912)

Bythoscopus kogotensis Matsumura, 1912: 305
Oncopsis juglans , Ishihara 1953: 21, misidentified (nec Matsumura 1912)
Oncopsis (Sispocnis) juglans , Anufriev 1967: 174, misidentified (nec Matsumura 1912)
Oncopsis (Sispocnis) kurentsovi Anufriev, 1977: 12 (synonym by Okudera 2014)
Pediopsoides (Sispocnis) juglans Anufriev and Emeljanov 1988: 78, misidentified (nec Matsumura 1912)

#### Material examined.

28 ♂♂ 29 ♀♀, CHINA: Shanxi Province, Lishan National Natural Reserve, 23–26.vii.2012, collected by Song Qiong-Zhang, Zhang Pei & Xing Dong-Liang; 1 ♀, CHINA: Shanxi Province, Xinzhou City, Ningwu County, Luyashan National Natural Reserve, 19.viii.2011, collected by Yu Xiao-Fei; 2 ♂♂ 1 ♀, CHINA: Sichuan Province, Ya’an City, Tianquan County, Erlang Mountain, Labahe, 25.vii.2012, collected by Fan Zhi-Hua & Li Hu; 2 ♀♀, CHINA: Sichuan Province, Ya’an City, Fengtongzhai National Natural Reserve, 1500 m, 01.viii.2005, collected by Zhou Zhong-Hui & Xu Fang-Ling; 1 ♀, CHINA: Sichuan Province, Erlang Mountain, 04.viii.2005, collected by Yang Zai-Hua; 1 ♂, CHINA: Shaanxi Province, Ankang City, Ningshan County, Huoditang, Linchang, 12.vii.2012, collected by Li Hu; 2 ♀♀, CHINA: Shaanxi Province, Mei County, Taibaishan National Natural Reserve, Haopingsi, 12.vii.2012, collected by Xu Shi-Yan; 1 ♀, CHINA: Shaanxi Province, Qingmuchuan National Natural Reserve, 18.viii.2010, collected by Li Hu & Fan Zhi-Hua; 1 ♂, CHINA: Anhui Province, Liuan City, Tiantangzhai National Natural Reserve, 950 m, 01.viii.2013, collected by Li Bin, Jiao Meng & Yu Xiao-Fei; 1 ♀, CHINA: Hubei Province, Shiyan City, Wudang Mountains, 13.vii.2013, collected by Li Hu; 1 ♀, CHINA: Hubei Province, Shennongjia National Natural Reserve, Guanmenshan, 19.vii.2013, collected by Xing Dong-Liang; 1 ♂, CHINA: Hubei Province, Shennongjia National Natural Reserve, Yazikou, 1850 m, 11.viii.1997, collected by Yang Mao-Fa; 1 ♂, CHINA: Hubei Province, Wufeng Tujia Autonomous County, Houhe National Natural Reserve, 22.vii.2013, collected by Chang Zhi-Min; 1 ♀, CHINA: Guizhou Province, Leigongshan National Natural Reserve, 10.vii.2010, collected by Long Jian-Kun; 1 ♂ 3 ♀♀, CHINA: Guizhou Province, Leigongshan National Natural Reserve, Light traping, 04–06.vii.2011, collected by Chang Zhi-Min & Zheng Wei-Bin; 2 ♀♀, CHINA: Jilin Province, Changbaishan National Natural Reserve, 24.vii.2011, collected by Jiao Meng; 2 ♂♂ 1 ♀, CHINA: Henan Province, Xinxiang City, Huixian City, 800 m, 12.vii.2002, collected by Chen Xiang-Sheng; 2 ♂♂, CHINA: Henan Province, Baiyunshan National Natural Reserve, 14–17.viii.2008, collected by Li Jian-Da; 2 ♀♀, CHINA: Ningxia Hui Autonomous Region, Liupan Mountains, 2050 m, 28–29.vii.2008, collected by Song Qiong-Zhang; 1 ♀, CHINA: Jilin Province, Changbaishan National Natural Reserve, Baihe, 13.viii.1996, collected by Li Zi-Zhong; 1 ♀, CHINA: Liaoning Province, Benxi City, Laotudingzi National Natural Reserve, 19–20.vii.2011, collected by Fan Zhi-Hua; 10 ♂♂ 7 ♀♀, CHINA: Hebei Province, Chengde City, Wulingshan National Natural Reserve, 09.viii.2011, collected by Li Hu, Jiao Meng, Fan Zhi-Hua, Yu Xiao-Fei, Liang Wen-Qin & Zhang Xin-Feng.

#### Distribution.

Widespread in China (Jilin, Liaoning, Zhejiang, Hebei, Henan, Shanxi, Shaanxi, Ningxia, Anhui, Hubei, Guizhou and Sichuan), Korea, southern part of Primorsky Krai of Russia and Japan.

#### Remarks.

The combined features of the shape of the pygofer articulated lobe and ventral process, the relatively simple dorsal connective, and the structure of the aedeagus separate P. (S.) kogotensis from other species.

### Pediopsoides (Sispocnis) rectus

Taxon classificationAnimaliaHemipteraCicadellidae

Li, Li & Dai sp. nov.

http://zoobank.org/5BA796B9-A9DB-4027-A391-401A8D1A24BA

Figs 27–37

#### Type material.

HOLOTYPE: ♂, CHINA: Sichuan Province, Tibetan Autonomous Prefecture of Garzê, Luding County, Minya Konka, Yajiageng, 3800 m above sea level, 13.viii.2015, collected by Zhan Hong-Ping. PARATYPE: 1 ♂, CHINA: Sichuan Province, Tibetan Autonomous Prefecture of Garzê, Xiangcheng County, Shagong Town, Dagen, 3500–3900 m above sea level, 15.viii.2015, collected by Zhan Hong-Ping.

#### Etymology.

The new specific epithet was derived from the Latin words “*rectus*” indicating that the aedeagal shaft is straight relatively.

#### Description.

**Figures 27–37.**
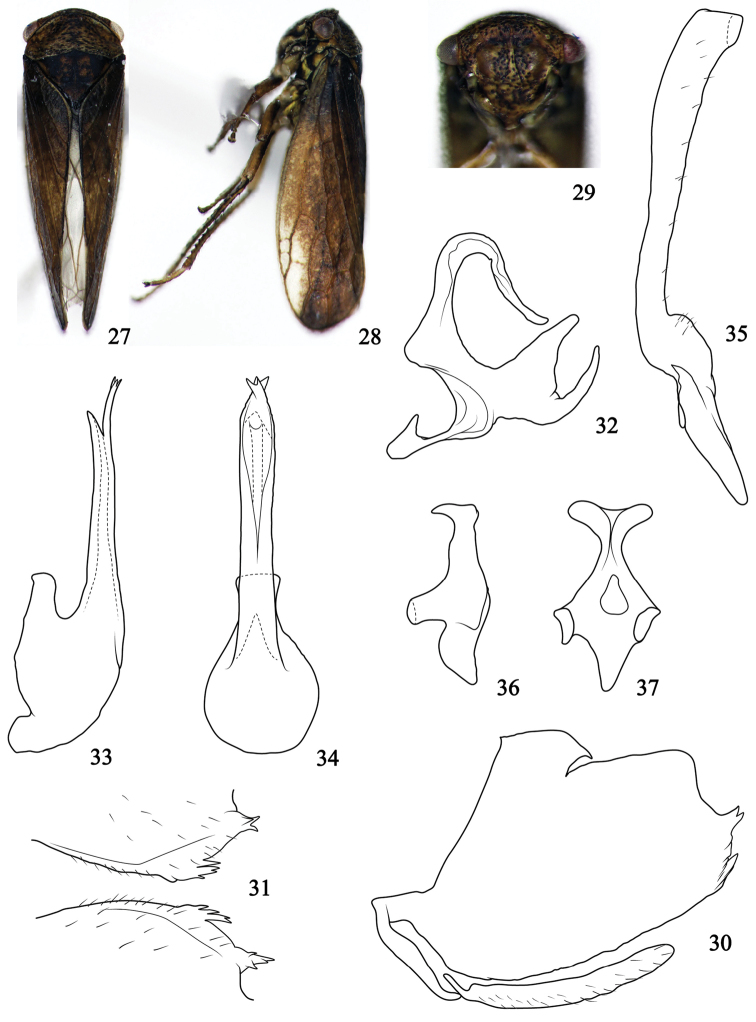
Pediopsoides (Sispocnis) rectus sp. nov. **27** male habitus, dorsal view **28** male habitus, lateral view **29** face **30** male pygofer and subgenital plate, lateral view **31** pygofer ventral processes, ventrocaudal view **32** dorsal connective, lateral view **33** aedeagus, later view **34** aedeagus, ventral view **35** style, dorsal view **36** connective, lateral view **37** connective, dorsal view.

*Body color*. Body background color (Figs 27, 28) chocolate, punctures on surface of head, face, pronotum and scutellum darker brown. Face (Fig. 29) brownish, eyes reddish brown on facial view and pale brown on dorsal view (Fig. 27). Scutellum (Fig. 27) darker brown, both lateral corners with black triangular spots. Forewing (Figs 27, 28) evenly brown except area around outer apical cell, venation darker.

*Body appearance*. Head (Fig. 27) short, and prominent forward, anterior margin slightly depressed near eyes, including eyes as wide as pronotum. Face (Fig. 29) covered distinct punctures; frons with weak carina and oblique striations, central part with two smooth inflated regions without any stripes or maculae; distance between ocelli nearly 4 times of that from ocellus to adjacent eye. Pronotum (Fig. 27) with obvious, intensively transverse striations and punctures, anterior margin round, and prominent frontally, posterior margin depressed in middle, 2.6 times broader than long. Scutellum (Fig. 27) surface granulose, mid-length 1.5 times that of pronotum. Forewing (Figs 27, 28) opaque, with three anteapical cells, veins prominent.

*Male genitalia*. Pygofer (Fig. 30) broad basally, dorsal margin incised and straight, distal part of ventral and caudal margins carries irregular small spine-like processes, and scattered setae. Subgenital plate (Fig. 30) slender, approximatively 0.65 times ventral margin of pygofer, marginated with setae. Aedeagus (Figs 33, 34) basally broad, dorsal apodeme well developed, shaft slender, and almost straight in lateral view, lateral margins parallel in ventral view, apex of ventral margin strongly expanded, and produced to bifid process which bifurcates again; gonopore apical. Dorsal connective (Fig. 32) in lateral aspect S-shaped, carries large bifurcate process from its inner ventral margin with both branches of equal length. Style (Fig. 35), stem stout and widened gradually to truncate apex, with marginal setae.

#### Measurement.

Body length (including tegmen): 4.8–4.9 mm.

#### Distribution.

China (Sichuan).

#### Remarks.

This new species somewhat resembles P. (S.) aomians with both sharing approximate color pattern and external body form more or less, but it can be distinguished from the latter and other known species of Pediopsoides (Sispocnis) by the different structures of the pygofer ventral processes, aedeagus and the dorsal connective.

### Pediopsoides (Sispocnis) sharmai

Taxon classificationAnimaliaHemipteraCicadellidae

Viraktamath, 1981

Pediopsoides (Sispocnis) sharmai Viraktamath, 1981: 308

#### Material examined.

None.

#### Distribution.

India.

#### Remarks.

Based on the original description by Viraktamath (1981), this species can be separated from other members of the subgenus mostly by the following features: the pygofer ventral margin has two spine-like processes distally, the tapered aedeagal shaft has an excavated distal margin in middle formed into a U-shaped in caudal view, and the dorsal connective has a bifid apex and caudally-directed triangular process from its inner ventral margin.

### Pediopsoides (Sispocnis) triangulus

Taxon classificationAnimaliaHemipteraCicadellidae

Li, Li & Dai sp. nov.

http://zoobank.org/4C4D305F-A4F3-415C-9D88-C5F7B63A3107

Figs 38–48

#### Type material.

HOLOTYPE: ♂, CHINA: Sichuan Province, Tibetan Autonomous Prefecture of Garzê, Luding County, Minya Konka, Yajiageng, 3800 m above sea level, 13.viii.2015, collected by Zhan Hong-Ping.

#### Etymology.

The new species name was derived from the Latin words “*triangulus*” referring to the triangular processes on the lateral margins of the aedeagal shaft.

#### Description.

**Figures 38–48.**
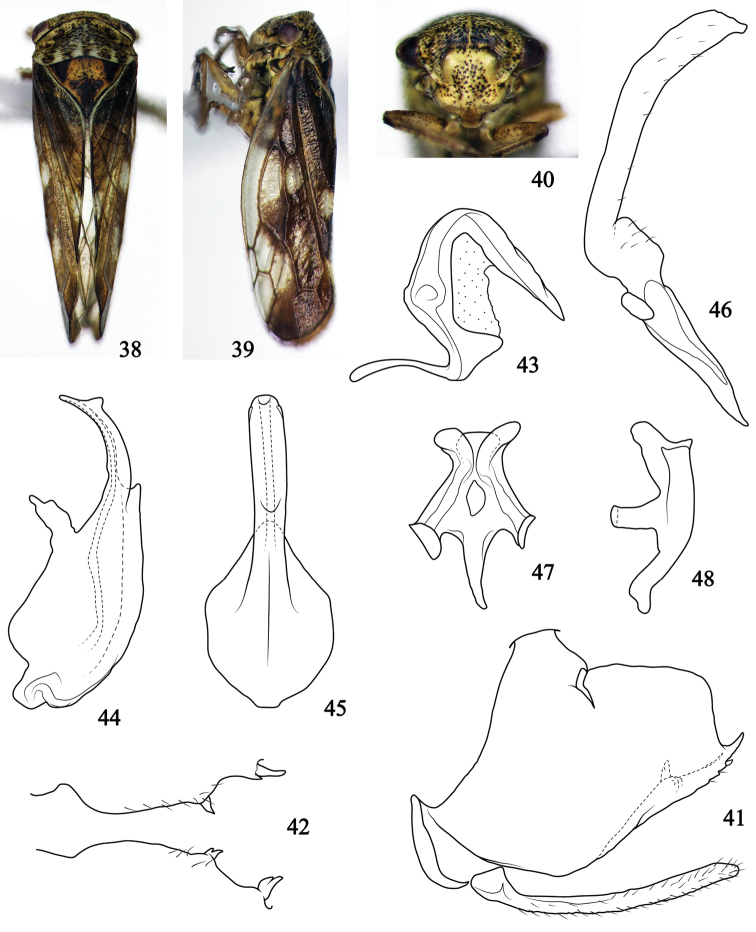
Pediopsoides (Sispocnis) triangulus sp. nov. **38** male habitus, dorsal view **39** male habitus, lateral view **40** face **41** male pygofer and subgenital plate, lateral view **42** pygofer ventral processes, ventrocaudal view **43** dorsal connective, lateral view **44** aedeagus, later view **45** aedeagus, ventral view **46** style, dorsal view **47** connective, dorsal view **48** connective, lateral view.

*Body color*. Background color (Figs 38, 39) yellow brown, punctures on body surface dark brown. Face (Fig. 40) yellowish, eyes dark brown, occasionally with reddish tinge. Pronotum (Fig. 38) with six pale yellowish subtriangular spots on posterior margin. Scutellum (Fig. 38) orange brown, both lateral corners with black triangular spots, and with dark posterior half. Forewing (Figs 38, 39) mainly pigmented by dark brown except transparent parts.

*Body appearance*. Head (Fig. 38) prominent frontally, clearly shorter medially than next to eyes, head including eyes as wide as pronotum. Face (Fig. 40) inflated in lateral view; frons with intensive punctures, oblique stripes and mid-carina; central part with n-shaped smooth region; ocelli closer to eyes, distance between them nearly 5 times of that from an ocellus to adjacent eye. Pronotum (Fig. 38) oblique forward and laterally, 2.7 times broader than long, with obvious, intensive and transverse striations and punctures, anterior area near eyes depressed, posterior margin excavated in middle. Scutellum (Fig. 38) surface granulose, and scattered with punctures, mid-length about 1.8 times that of pronotum. Forewing (Figs 38, 39) with three anteapical cells, veins prominent.

*Male genitalia*. Pygofer (Fig. 41) broad basally, with incised dorsal and caudal margins, distal half of each ventral margin produced into two small spine-like processes which sometimes bifid. Subgenital plate (Fig. 41) narrow and elongate, nearly as long as that of ventral margin of pygofer, and with marginal setae. Aedeagus (Figs 44, 45) basally broad, with strongly developed dorsal apodeme and preatrium, shaft short, clearly narrower than basis in lateral view, lateral margins parallel in ventral view, subapex with small triangular processes directed caudally on lateral margins, apex round; gonopore apical. Dorsal connective (Fig. 43), in lateral aspect, S-shaped, process from inner ventral margin short, and horn-like. Style (Fig. 46), stem bent dorsolaterally, apex with small expansion. Connective (Figs 47, 48) typical.

#### Measurement.

Body length (including tegmen): 4.8 mm.

#### Distribution.

China (Sichuan).

#### Remarks.

The new species is similar to P. (S.) aomians in the pattern of body coloration, and somewhat similar to P. (S.) dilatus in male features, but can be distinguished from both by the distal half of the pygofer ventral margin with two small spine-like processes which are sometimes bifid, different shapes of the aedeagus and the dorsal connective.

##### Key to species of Pediopsoides (Sispocnis) based on male genitalia

**Table:** 

1	Pygofer with articulated lobe, and one definite process inturned from ventral margin; dorsal connective relatively small, without process from inner ventral margin; aedeagal shaft with clear lateral expansions subapically	**P. (S.) kogotensis**
–	Pygofer lobe without obvious suture on pygofer side, and with more than one processes or teeth on ventral margin; dorsal connective large, with various process from inner ventral margin; aedeagal shaft not as above	**2**
2	Pygofer ventral margin with series of teeth on distal half; aedeagal shaft narrowed basally, and gradually broadened to end in ventral view	**P. (S.) dilatus**
–	Pygofer ventral margin with bifid or multifid processes on distal half; aedeagal shaft broad basally, or with parallel margins in ventral view	**3**
3	Dorsal connective with unbranched process from its inner ventral margin	**4**
–	Dorsal connective with branched process from its inner ventral margin	**5**
4	Aedeagal shaft with small triangular processes subapically on lateral margins, and round apex; dorsal connective without bifid apex	**P. (S.) triangulus sp. nov**.
–	Aedeagal shaft without process on lateral margins, but with U-shaped apex; dorsal connective with bifid apex	**P. (S.) sharmai**
5	Pygofer ventral margin with large and figure-like multifid (2–4) processes at distal half; aedeagus stout, and bent dorsally, apex of shaft expanded lateroapically	**P. (S.) aomians**
–	Pygofer ventral margin with small and short multifid (more than 5) processes at distal half; aedeagal shaft slender, straight, apex of shaft only expanded apically	**P. (S.) rectus sp. nov.**

## Supplementary Material

XML Treatment for Pediopsoides

XML Treatment for Pediopsoides (Sispocnis)

XML Treatment for Pediopsoides (Sispocnis) aomians

XML Treatment for Pediopsoides (Sispocnis) dilatus

XML Treatment for Pediopsoides (Sispocnis) kogotensis

XML Treatment for Pediopsoides (Sispocnis) rectus

XML Treatment for Pediopsoides (Sispocnis) sharmai

XML Treatment for Pediopsoides (Sispocnis) triangulus
